# A policy-driven mental health and psychosocial support in African Union Peace Support Operations: the way forward in improving the psychosocial wellbeing of peace support personnel

**DOI:** 10.3389/frhs.2025.1465236

**Published:** 2025-07-28

**Authors:** Joana Afful Larry-Afutu, Kenneth Abotsi

**Affiliations:** ^1^ Department of Psychology, University of Ghana, Accra, Ghana; ^2^ Peace Support Operations Division, Political Affairs, Peace and Security Department, African Union Commission, Addis Ababa, Ethiopia

**Keywords:** African Union, Mental Health and Psychosocial Support (MHPSS), Peace Support Operations, mental health, psychosocial problems, wellbeing, policy

## Abstract

**Background:**

To foster peace and security within Africa, the African Union Peace Support Operations (AUPSO) Division deploy personnel to volatile areas, predisposing them to physical and psychosocial problems. Though a lot is done to safeguard their physical health, their psychosocial problems have been relegated, predisposing them to mental health problems.

**Analysis:**

To address these mental health and psychosocial problems, the African Union, in collaboration with Kofi Annan International Peacekeeping Training Centre, Ghana developed and implemented a Mental Health and Psychosocial Support (MHPSS) course for AUPSO personnel. Implementing MHPSS in mission areas is bedeviled with challenges, because there are no policy guidelines for MHPSS in PSOs.

**Policy options:**

At the behest of WHO's Action Plan 2013–2020 which encourages institutions and organizations to prioritize mental health of personnel, the United Nations adopted a mental health and well-being strategy to enhance the psychosocial wellbeing of its mission's personnel in 2018.

**Conclusion:**

Feasibly, developing a MHPSS policy for PSOs would enhance the implementation of MHPSS programs in missionary areas to ensure that personnel are in a good psychosocial state to fulfil their missions’ mandate.

## Introduction

Like others, the African continent has been wrought with several conflicts from political transitioning, protecting territorial borders, inter-ethnic and/or inter-state disturbances and many more (1). To foster peace and security within Africa, the African Union Peace Support Operations Division is mandated to plan, launch, sustain, monitor and liquidate all peace support operations in the sub-region (2). The AU relies on uniformed and non-uniformed personnel in implementing these activities. These personnel are often deployed to volatile environments, placing on them various demands for adjustments, the failure of which leads to many physical and mental health problems (3). Over the years, the mental health of PSO personnel, which is essential in fulfilling the mandate of PSOs, has not been given serious attention in planning and implementing PSOs. With emerging trends in global peacekeeping and peace enforcement, it has become necessary for stakeholders of AU PSOs to garner insight into how the nature of stressors and traumatic incidence in the PSO environment predispose PSO personnel to different degrees of mental health challenges, to initiate interventions aimed at reducing the impact of these stressors on the fulfilment of the PSO mandate (4).

### African Union Peace Support Operations

The African Union Doctrine on PSO (5) outlines the core principles, practices and approaches that guide AU PSO, within the framework of African Peace and Security Architecture (APSA), as part of AU's objective to promote peace, security and stability on the continent. According to this Doctrine, Peace Support Operations refer to “a generally multinational, multifunctional and multidimensional operation, mandated and deployed by an international, regional or cross regional arrangement to restore or maintain peace within a specific area of operations.” The multidimensionality of the PSO requires that different categories of personnel from the military, police and civilians work together in the implementation of the mission's mandate. As well, the multifunctional and multidisciplinary nature of PSO operations requires that PSO personnel utilize different processes, modalities and approaches to support its objectives and mandate, including but not limited to dialogue and reconciliation, security initiatives as well as institutional capacity building and peacebuilding measures to facilitate implementation and achievement of its mandate.

In a typical AU PSO environment, social cohesion and state institutions have usually collapsed, and law and order have broken down, leading to several human rights violations, war crimes, crimes against humanity, genocide and many more atrocities. These occurrences expose both the deployed and community members to several psychosocial challenges that pre-dispose them to mental health problems.

### Mental health of PSO personnel

The World Health Organization's (WHO) systematic review of 129 studies in 39 countries established that:

a) Nearly all people who are affected by traumatic incidents will experience psychological distress.
b) One person in five (22%) living in conflict zones is estimated to have depression, anxiety, post-traumatic stress disorder, bipolar disorder or schizophrenia.
c) People with profound mental health problems become vulnerable when there is trauma and need access to urgent mental health care.
d) For these reasons, there are international guidelines which recommend some level of mental health care services as part of health response to all affected people in conflict areas (6).

In view of these challenges, the Kofi Annan International Peacekeeping Training Centre (KAIPTC), Ghana in collaboration with AU PSO Division and GIZ, developed and implemented a course on Mental Health and Psychosocial Support (MHPSS) for AU PSO personnel in 2020 and 2021 respectively. The course runs every year since then with an E-learning version, and during some of these training sessions, PSO personnel in group discussions asserted that, in carrying out their mandates in the mission areas, they encounter numerous stressful and potentially traumatic events before, during and after deployment as outlined below:

### Pre-deployment

Stressors identified at this stage include sudden changes in lifestyle and routine due to pre-deployment preparations, separation from family to training camps, exhaustion from pre-deployment training, uncertainty about operational and geographical conditions in deployment areas and uncertainty about the duration of the mission. These stressors have potential mental health implications.

### Deployment

PSO personnel explained that during deployment, they are confronted with stressors and traumatic events that are typical in conflict zones. For instance, constant witness of death of indigenes and fellow peacekeepers, evacuation of dead bodies for burial, sudden explosions of landmines, being fired upon, intermittent attacks from rebels and atrocities commited against some peacekeepers and indigenes could leave indellible marks on their minds for months. Again, they are confronted with the challenge of operating under restrictive rules of engagement, which often lead to role conflict and ambiguity with regards to appropriate action to be taken under situations of threat to themselves or others. They also experience a sense of helplessness if they are unable to reduce people's suffering and improve their safety adequately. These notwithstanding, they also contend with restriction of movement, limited variety of food to choose from, boredom with routine and uncertainties of survival on daily basis.

### Post-deployment

They indicated that, leaving the PSO environment does not preclude the impact of accumulated stressors and trauma associated with the PSO environment. They contend with readjusting to schedules and routines both at work and home, insomnia, racing thoughts about the deployment experiences, irritability and anxiety. They added that sometimes, some of them pick up unacceptable behaviours such as excessive alcohol intake, aggression and withdrawing from loved ones as a way of coping.

### Analysis

#### Opportunities for implementing MHPSS or Opportunities for implementing Mental Health and Psychosocial Support (MHPSS)

Since PSOs have been found to impact negatively on the mental health of personnel and the members of the communities into which they are deployed, and consequently the successful execution of their mandated tasks, it is important to proactively address mental health and psychosocial challenges associated with current and future deployments.

Mental health issues have been relegated in many societies due to the stigma associated with it. However, sensitization due to current occurrences globally, like COVID 19 and associated problems have underscored the need to be mindful of mental health issues. Furthermore, in line with the WHO's Action Plan 2013–2020 (7), several multinational institutions and organizations have incorporated psychosocial support into their operations for personnel who may be having a hard time adjusting to work-related stressors. Thus, AU PSOs' need to be conscious about promoting the wellbeing of PSO personnel, preventing them from developing mental disorders, providing care, and enhancing recovery where appropriate.

In 2018, the United Nations adopted Mental Health and Well-Being Strategy which prioritized mental well-being for its missions' personnel and incorporated activities and procedures for addressing mental health and psychosocial problems in preparing and deploying for missions (4). At its 9229th meeting in December 2022, the UN Security Council unanimously adopted a resolution S/RES/2668(2022) (8), stressing the need to raise awareness of the importance of MHPSS for United Nations peace operations personnel. It encouraged troop- and police-contributing countries, including member states and the Secretariat to provide mental health services to support personnel during the pre-deployment, deployment and post-deployment stages with adequate mental health and psychosocial support services.

In the African Union Missions, these institutional systems have not yet been established, making the implementation of MHPSS for AU PSO personnel an arduous task. The nature of the AU PSO operations and the experiences of personnel creates an avenue for tailor-made interventions and implementation for AU PSO personnel aimed at mitigating the impact of PSO experiences on the mental health of personnel.

#### Challenges for implementing MHPSS

There have been some admissions that mental health and psychosocial problems prevail in the AU PSO environment, thus there is the need to ensure that steps are taken to provide MHPSS to AU PSO personnel. Though there have been some attempts by the AU to incorporate MHPSS in the activities of PSOs, these attempts are met with some challenges as follows.

a) The absence of a continent-wide policy that responds to the mental health and psychosocial needs of PSO personnel render leaders and commanders who are educated on MHPSS issues helpless, as they have solutions but no resources to implement them.
b) Lack of commitment by leadership of PSOs to establish robust mechanisms for providing MHPSS within missions due to low or no budgetary allocations for recruiting professionals to provide such specialized services for personnel.
c) Stigmatization of mental health issues despite growing recognition of its high prevalence among PSO personnel discourage them from reaching out for available support. Besides, strong gender norms discourage men particularly from accessing available support.
d) The institutional culture of the military for instance has made “fitness for hardship” a core expectation for PSO personnel and this idea discourages soldiers from acknowledging mental health issues and seeking mental healthcare.
e) The experts trained to address mental health and psychosocial problems in the continent are few.
f) Research, an essential tool for gaining insight into PSO MHPSS issues, and guiding interventions is not encouraging.

With these challenges, there is the need for a conscious effort and commitment towards the development and implementation of interventions to prevent and manage AU PSO MHPSS issues.

### Policy options

The following are recommendations towards the implementation of AU PSO context MHPSS to mitigate the impact of PSO stressors on mental health of PSO personnel.

a) Extensive stakeholder sensitization of mental health and psychosocial issues in PSOs geared towards reduction of stigma associated with mental health and promoting help seeking to reduce the incidence of mental health problems in PSOs.
b) Policy driven initiatives focusing on prevention, early recognition of symptoms and easy access to treatments and psychosocial support must be a high priority for PSOs. These initiatives should be aimed at improving their mental health before, during and after deployments. A policy driven intervention would also enhance mechanisms for providing MHPSS through the integration and institutionalisation of mental health services in planning, launching, sustaining, monitoring and liquidating PSOs. It would also clarify the roles and responsibilities of various stakeholders and harmonize procedures and mechanisms for assessing MHPSS services. The policy could also address issues relating to multiple deployments to similar traumatic environments to prevent re-traumatisation.
c) Mental health experts could be hired to train potential PSO personnel with basic interventions in MHPSS, so that in the PSO environment, they could support peers with basic psychosocial support while referring people with mental health difficulties to designated experts.
d) Encouraging scientific research into the mental health of AU PSO personnel would also be appropriate in monitoring the nature, degree and statistics of mental health problems of AU PSO personnel. This data would be useful in designing tailor-made interventions for AU PSO personnel.
e) Some proposed initiatives at the various stages of deployments have also been recommended:

### Pre-deployment initiatives

Troop/Police Contributing Countries (TCCs/PCCs) could initiate a mandatory professional-led psychological assessment, education, and support as part of preparations towards deployment by:

• Undertaking psychological evaluation of personnel earmarked for deployment as part of pre-deployment medical screening. Results from these screenings could be used as a baseline of the mental health state of troops, based on which specific interventions are put in place to address specific psychosocial needs of troops. Results should not be used to preclude staff from being deployed as this could lead to stigmatization. Nevertheless, results could be used as an opportunity to sensitize personnel on stressors, mental health challenges abd available resources for coping and maintaining their mental well-being.
• Pre-deployment training should mandatorily include a mental health module which would counter prejudice, stigma and discrimination against mental health, address specific stressors encountered in the field, share well-being and stress-management techniques, including how to prevent, identify and cope with stress, trauma, and other mental health issues, and provide information on support systems in the country of origin and in the mission area. Personnel earmarked and/or appointed to senior leadership positions in missions should also be trained on how to cater for staff wellbeing and mental health.
• Structures could also be put in place ahead of deployment to address stressful situations. For instance, stress-relieving social activities, sports, and recreation at all levels of a PSO could be planned for prior to missions.
• Training of medical and religious personnel nominated for PSO to recognize and manage signs and symptoms of stress within the PSO environment, as they are usually the go to personnel in times of distress in the mission area.
• Nominees for deployment should have access to professional counselling prior to departure as may be required.

### Deployment initiatives

Deployment initiatives could involve establishing and managing professional help for PSO personnel during deployment. The responsibility for this mostly lies with the Troop/Police contributing Countries (TCCs/PCCs), the mission itself and other agencies in the mission area who specialise in providing mental health and psychosocial support.

In the light of this, the following could be considered:

• Continuous education of personnel on in-mission structures for accessing psychosocial support
• Deployment of psychologists/counsellors within the mission
• Mandatory screening of all deployed personnel at least midway through the missions or end of mission
• Mental health training for personnel including training on detecting signs of stress and coping.
• Providing regular avenues for relieving stress. These could include rest and recreation (R&R), sports, music, dancing etc. There is consensus that R&R is crucial to the mental health of staff. However, if not enforced consistently, some PSO personnel may forfeit their R&R and stay in the field for long periods of time. Beyond R&R, there should be staff counselors in missions and emergency structures that can be activated when required.
• Providing peer support to personnel

### Post-deployment initiatives

At the post-deployment phase, provision should be made for a transitional phase between the operational theatre and homecoming for deployed personnel. This transition phase can serve as a period or re-evaluation of personnel's mental health state and a catalyst for providing support to personnel to adequately prepare and cope with typical homecoming challenges.

Post-mission psychosocial support programmes should be aimed at:

• Facilitating and easing the transition from combat-life to non-combat life: reducing the stress associated with reintegration and readjustment into work and family life. For instance, disembarkation leave could be mandatory and guided.
• Promoting mental wellbeing by promoting rest, relaxation and recreation activities.
• Sensitization on potential post-deployment mental health and psychosocial problems, coping and available services.
• Psychological debriefing and post-deployment psychological assessment aimed at early detection of mental health symptoms and providing avenues for counselling and mental health care.
• Empowering post-deployed soldiers as mental health peer support agents to share their success stories with soldiers who are nominated for deployment, identify mental health symptoms and render peer support or refer peers to experts where necessary.
• Sensitize the family of post-deployed soldiers on mental health and potential implications of deployment on the mental health of post-deployed soldiers and support services available.

## Conclusion

The PSO environment negatively impacts the mental health of PSO personnel and the members of the communities into which they are deployed, and the successful execution of their mandated tasks. Consequently, the need for the AU to design a continent-wide policy that addresses the mental health and psychosocial challenges associated with current and future PSOs comprehensively is not an option but an imperative.
